# Desmethylbellidifolin From *Gentianella acuta* Ameliorate TNBS-Induced Ulcerative Colitis Through Antispasmodic Effect and Anti-Inflammation

**DOI:** 10.3389/fphar.2019.01104

**Published:** 2019-09-20

**Authors:** Yajuan Ni, Mengyang Liu, Haiyang Yu, Yue Chen, Yanxia Liu, Suyile Chen, Jingya Ruan, Alatengchulu Da, Yi Zhang, Tao Wang

**Affiliations:** ^1^Institute of Traditional Chinese Medicine, Tianjin University of Traditional Chinese Medicine, Tianjin, China; ^2^Tianjin State Key Laboratory of Modern Chinese Medicine, Tianjin University of Traditional Chinese Medicine, Tianjin, China; ^3^Key Laboratory of Pharmacology of Traditional Chinese Medical Formulae, Tianjin University of Traditional Chinese Medicine, Ministry of Education, Tianjin, China; ^4^Alxa League Mongolian Medical Hospital, Bayanhaote, China

**Keywords:** *Gentianella acuta*, ulcerative colitis, desmethylbellidifolin, inflammation, intestine contraction

## Abstract

Desmethylbellidifolin (DMB) is a natural xanthone extracted from *Gentianella acuta*, which is used as the antidiarrhea drug in traditional Mongolian medicines. It remains unknown whether DMB can ameliorate ulcerative colitis (UC). In this study, trinitrobenzenesulfonic acid (TNBS)-induced colitis rats were treated with *G. acuta* extract (GAE) or DMB for 10 days. Body weight, food and water intake, rectal bleeding score, diarrhea score, and histopathological parameters were measured. Rat colon were collected to determine myeloperoxidase, nitric oxide levels, and inflammatory cytokines expression. In addition, the role of DMB on lipopolysaccharide stimulated RAW264.7 cell inflammatory response and intestine smooth muscle contraction was determined. The results showed that GAE and DMB treatment could significantly alleviate TNBS-induced UC. Colon morphological alteration, nitric oxide level, and inflammatory cytokines level, such as nitric oxide synthase, interleukin-6, tumor necrosis factor-α, and cyclooxygenase-2, were decreased. In addition, DMB attenuated lipopolysaccharide-induced nitric oxide release and proinflammatory cytokine expression in RAW264.7 cells. In isolated mice intestinal tissue, DMB also reduced the intestine smooth muscle spontaneous contraction and inhibited KCl, acetylcholine, BaCl_2_, or histamine-induced intestine smooth muscle active tension, while the active frequency was unaffected. Our results demonstrated that GAE and its active constituent DMB could inhibit TNBS-induced UC, reducing inflammatory response and alleviate colon muscle spasm, suggesting that DMB may be a good candidate for subsequent development as a multitargeting drug for UC treatment.

## Introduction

Ulcerative colitis (UC) is a chronic inflammatory bowel disease with ulcers of the colon. The symptoms of UC are abdominal pain and uncontrolled diarrhea mixed with blood. Although UC is not fatal, the long-term abdominal uncomfortable lowers the life quality of the patient. Epidemiological investigation revealed that the global prevalence of UC is increasing at the turn of the twenty-first century (Kaplan and Ng, 2017), and about 1–2 of every 1,000 people in developed countries are affected with UC (Blumberg et al., 2011). Age distribution analysis indicates that 10–20% of UC cases are diagnosed in older adults over 60 years of age (Katz and Pardi, 2011). Although there is an effectively alleviative method to moderating UC symptoms through diet changes, patient may still require pharmacotherapy when long-term diarrhea cannot be controlled by life style modification (Verma et al., 2000; Herfarth et al., 2014).

The cause of UC is not completely understood. Recently, its pathogenesis has been considered as environmental influences, imbalanced microbiomes, genetic variations, and disturbances in the innate and adaptive immune responses (Schirbel and Fiocchi, 2010). The increased enteric mucosal chronic inflammatory cells, immune cell activation, and inflammatory cytokine release are the main pathological features of UC, which leads to intestinal inflammation and abnormal intestinal motility (Fiocchi, 1998; Swidsinski et al., 2002). In patients with active colitis, the increased intestinal contraction is closely related to mucosal inflammation and ulceration (Loening-Baucke et al., 1989). Clinically, there is no single ideal therapy for UC. Different kinds of medicines are used to halt or ease UC symptoms, such as sulfasalazine to treat inflammation or moderate pain, loperamide to treat diarrhea, and alosetron to manage bowel movements (Singh et al., 2018). However, these single-target antagonists or inhibitors could only control part of the UC symptoms with low efficacy. A combination of these medications may have better effects, but it also causes inconvenience and increases the possibility of noncompliance.

*Gentianella acuta* (Michx.) Hulten, belonging to the family Gentianaceae, is an herbal medicine used as antidiarrhea in traditional Chinese medicines, which suggest that it may also exert some beneficial effects for UC treatment. Our previous study showed that its compound, desmethylbellidifolin (also named as 1,3,5,8-tetrahydroxyxanthone, DMB), a natural xanthone, significantly reduced the isolated intestine contraction tension (Liu et al., 2016b). In this study, the effect of *G. acuta* extract (GAE) and DMB on UC was investigated using 2,4,6-trinitrobenzenesulfonic acid (TNBS) acid-induced rat colitis model. Lipopolysaccharides (LPS) stimulated RAW264.7 cells and isolated mice intestinal tissue. We determined if DMB could protect UC by inhibiting inflammatory factors expression and gut motility and tried to disclose the underlying mechanisms of DMB in the treatment of UC.

## Materials and Methods

### Materials

The whole plants of *G. acuta* (Michx.) Hulten were collected from Alxa Youqi, Inner Mongolia Autonomous region, China, and identified by Dr. Chunhong Zhang (Alashan Mongolian Hospital). The voucher specimen was deposited at Herbarium, Institute of Botany, Chinese Academy of Sciences, Beijing, China (Voucher Number: 02293698).

Balsalazide sodium tablets (BST) were purchased from Zhendong Anter Pharmaceutical, Ltd (Shanxi, China). TNBS and L-N6-1-iminoethyl-lysine were purchased from Sigma-Aldrich LLC. (St. Louis, MO, USA). Bicinchoninic acid protein assay kit was from Thermo Scientific (Waltham, MA, USA). Rabbit anti-β-actin, anticyclooxygenase-2 (anti-COX-2), antinitric oxide synthase (anti-iNOS), and antitumor necrosis factor-α (anti-TNF-α) antibodies were obtained from Abcam plc. (Cambridge, MA, USA). All other chemicals were purchased from Sigma-Aldrich (St. Louis, MO) except as indicated.

### GAE Preparation and High-Performance Liquid Chromatography Analysis

The whole plants of *G. acuta* (3.0 kg) were ground and extracted with 70% ethanol–water under reflux for two times. The combined extracting solution was concentrated in vacuum to afford a 70% ethanol–water extract (GAE) (868.5 g). DMB was isolated from *G. acuta* as in our previous report (Liu et al., 2016b), the purity of which was more than 95% as determined by high-performance liquid chromatography (HPLC) method. DMB content in GAE was determined by HPLC method as describe as the literature (Liu et al., 2015).

### Animals

All animal experiments were approved by Tianjin University of Traditional Chinese Medicine Committee on Use and Care of Animals (TCM-LAEC20170037). Sprague–Dawley rats (weight = 180 ± 10 g) and Kunming mice (weight = 25 ± 5 g) were purchased from Vital River Laboratory Animal Technology Co. Ltd., (Beijing China). Both of them were acclimated for 1 week before the experiments. All animals were allowed free access to a standard diet and drink *ad libitum* and adapted to the experimental conditions at 22 ± 2°C, and humidity 60 ± 5% with a fixed 12-h artificial light period.

### TNBS-Induced UC Rats

After 1 week of adaption, male Sprague–Dawley rats were randomized into six groups (*n* = 8). Except in the normal group, colitis was induced by TNBS according to the literature report method with some modifications (Morris et al., 1989). Briefly, after fasting for 16 h, rats were lightly anesthetized with anhydrous diethyl ether. Subsequently, a 300-μl TNBS solution [5% (*w*/*v*) in 50% ethanol] was injected through the anus using a cannula that was introduced 8 cm in depth. Normal group was treated with the same volume of phosphate-buffered saline (PBS). After 24 h, DMB or GAE was administrated orally in 5% gum acacia, while the BST and control groups received the same volume of 5% acacia water solution with or without 1 g/kg BST. The treatments were continued for 10 consecutive days. Body weight (BW), food weight, and water volume of animals were recorded daily during the experiment. Damage score was scored for macroscopically visible damage on a 0–10 scale. Rectal bleeding and diarrhea were monitored by manual analyses of the level of blood and water content in the feces. Three persons unaware of the experiment independently performed foregoing analyses. The disease activity index was calculated by assigning well-established and validated scores. Briefly, the following parameters were used for calculation: (a) weight loss (0 point = none, 1 point = 1–5% weight loss, 2 points = 5–10% weight loss, 3 points = 10–15% weight loss and 4 points more than 15% weight loss), (b) diarrhea (0 points = normal, 2 points = loose stools, 4 points = watery diarrhea), (c) bleeding (0 points = no bleeding, 2 points = slight bleeding, 4 points = gross bleeding) (Alex et al., 2009). At the end of final administration, rats were fasted for 24 h and killed with a lethal dose of sodium pentobarbital (100 mg/kg, i.p.). Colon was quickly removed, opened longitudinally, and gently washed with ice-cold PBS. Then, colon weight and length were measured under a constant load. Macroscopic assessment of the colitis grade was scored according to a previously reported scoring system (Bell et al., 1995). All the colon was frozen in liquid nitrogen and stored at −80°C until analysis.

Colon samples were prepared for 10% homogenate in saline. NO level, myeloperoxidase (MPO) activity, and glutathione content were determined using commercial kits (BioSino Bio-technology and Science Inc., China). Protein concentration was measured quantitatively using bicinchoninic acid protein assay kit.

### Histopathology of Colon Tissues

The colon tissues of rats were removed, fixed with 4% paraformaldehyde in PBS, and then decalcified for 10 days with ethylenediaminetetraacetic acid and embedded in paraffin for histological analysis. The paraffin sections were stained with hematoxylin and eosin (HE), and photographed with Axio Imager D2 (Zeiss, Oberkochen, Germany) for conventional morphological evaluation. Microscopic score was calculated as in literature report (Yang et al., 2016). The microscopic score was evaluated on a 0–10 scale by a pathologist observer, who was unaware of the experiment groups.

### Isolated Intestinal Tissue Preparation and Isometric Measurements

Mice were euthanized with CO_2_. Intestinal tissues (including jejunum and ileum) were harvested quickly and cut transversely into 1-cm sections. The tissue section was mounted longitudinally, and the contraction of longitudinal smooth muscle was recorded following previously reported methods (Liu et al., 2016b). Briefly, the lower end of the intestine section was tied with a holder at the bottom of the organ bath, while the upper end was connected to an isometric force transducer with a silk-braided nonabsorbable suture (Johnson & Johnson Medical (China) Ltd., Shanghai, China) for recording mechanical activity. Intestine contractions were recorded using the Power Lab system and Chart 7 software (AD instrument Ltd., Australia). The Maxwell bath was filled with 10 ml of Tyrode’s solution (1 L contains NaCl 8.0 g, CaCl_2_ 0.2 g, KCl 0.2 g, MgCl_2_ 0.1 g, NaHCO_3_ 1.0 g, KH_2_PO_4_ 0.05 g, glucose 1.0 g, pH 7.4) and maintained at a constant temperature (37.0 ± 0.5°C) and bubbled with 95% O_2_ and 5% CO_2_ gas. The intestine sections were allowed to equilibrate for 30 min before the experiment started. During the equilibration period, the section was rinsed every 30 min with Tyrode’s solution, and the basal tension was maintained. Acetylcholine (ACh, 0.1 μM), histamine (HA, 10 mg/l), BaCl_2_ (50 mg/l), and KCl (40 mM) were used for mechanism researches, respectively.

### RAW264.7 Macrophage Cell Culture and LPS-Induced Nitric Oxide Production

RAW264.7 cells, a murine macrophage cell line (IBMS, CAMS/PUMC, Beijing China), were cultured in Dulbecco’s modified Eagle’s medium high glucose (Hyclone, Logan, UT, USA) medium with 10% fetal bovine serum (HyClone, Logan, UT, USA), 100 U/ml penicillin, and 100 μg/ml streptomycin in a humidified atmosphere containing 5% CO_2_ at 37°C. Cells were plated in a six-well plate at a density of 4 × 10^5^ cells/well for further experiments.

The concentration of NO in culture supernatants was determined by measuring the nitrite levels using Griess reagent. RAW264.7 cells were pretreated with DMB at different concentrations for 1 h and then stimulated with LPS (500 ng/ml) for 24 h. After incubation, aliquots of 100 μl of each culture medium was mixed with an equal volume of Griess reagent (St. Louise, MO). Nitrite levels were determined using an ELISA plate reader at 540 nm, and concentrations were calculated by reference to a NaNO_2_ standard calibration curve.

### Real-Time RT-PCR Analysis

RNA isolation of tissue and cells, complementary DNA synthesis and real-time PCR analysis were performed as described previously (Zhang et al., 2013). The primer sequences used for real-time PCR were synthesized by Dingguo Bio Co. Ltd, Shanghai, China (**Table 1**). Results were presented as levels of expression relative to those of controls after normalization to glyceraldehyde 3-phosphate dehydrogenase using the 2^−ΔΔCT^ methods. Analysis was carried out in triplicates.

**Table 1:** Sequences of the primers for real-time RT-PCR analysis.

Species	Gene	Primer sequence	
Rat	*COX-1*	F: AAGGGAAGAAGCAGTTACCAG	R: GCAAAGAAAGCAAACAAGACG
	*COX-2*	F: TCGGAGGAGAAGTGGGTTTTAG	R: TTGATGGTGGCTGTCTTGGTAGG
	*IL-6*	F: TCTTGGGACTGATGTTGTTG	R: ACTGGTCTGTTGTGGGTGGT
	*TNF-α*	F: GATGTGGAACTGGCAGAGGAG	R: CACGAGCAGGAATGAGAAGAG
	*iNOS*	F: TTGGAGCGAGTTGTGGATTGTT	R: TAGGTGAGGGCTTGCCTGAGTG
	*eNOS*	F: CACGAGGACATTTTCGGACT	R: CCAGGTGTTTCTTGGGTAGG
	*GAPDH*	F: TGAGGCCGGTGCTGAGTATGT	R: CAGTCTTCTGGGTGGCAGTGA
Mouse	*COX-1*	F: AGAAGGAGATGGCTGCTGAGTT	R: GTGAGGCTGTGTTGACAAGGTT
	*COX-2*	F: CTTCCTCCTGTGCCTGATGAT	R: GCCCTCGCTTATGATCTGTCT
	*IL-6*	F: AGACTTCCATCCAGTTGCCTT	R: TTCTCATTTCCACGATTTCCC
	*TNF-α*	F: GCCCAGACCCTCACACTCAGA	R: TAGACAAGGTACAACCCATCG
	*iNOS*	F: CTTGGAGCGAGTTGTGGATTGT	R: AGGTGAGGGCTTGGCTGAGTGA
	*eNOS*	F: ATTTCCTGTCCCCTGCCTTCC	R: GTTGCCTTCACACGCTTCGCC
	*GAPDH*	F: AACTTTGGCATTGTGGAAGG	R: GGATGCAGGGATGATGTTCT

### Western Blotting Analysis

Protein isolation and Western blotting were performed as described previously (Liu et al., 2016a). Briefly, equal amounts of proteins (40–80 μg) were resolved by 8–12% sodium dodecyl sulphate–polyacrylamide gel electrophoresis and transferred to polyvinylidene difluoride membranes (Millipore, Bedford, MA, USA). The normal proteins blots were blocked with Quickblock TM Western blocking buffer (Beyotime Institute of Biotechnology, Nanjing, China). The membranes were incubated overnight at 4°C with primary antibodies. The blots were washed three times with Tris-buffered saline with Tween 20 (TBST, Beijing Solarbio Science & Technology Co. Ltd., Beijing, China) and incubated with horseradish peroxidase conjugated secondary antibody for 1 h. Blots were washed three times with TBST. The transferred proteins were visualized with ChemiDoc MP Imaging System (Bio-Rad, Hercules, CA, USA).

### Statistical Analysis

Values are expressed as mean ± SD. All the grouped data were statistically performed with SPSS 11.0. Significant differences between means were evaluated by one-way analysis of variance (ANOVA) and Tukey’s studentized range test was used for *post hoc* evaluations. The differences were considered significant at *P* < 0.05.

## Results

### DMB Content in GAE

**Figure 1:**
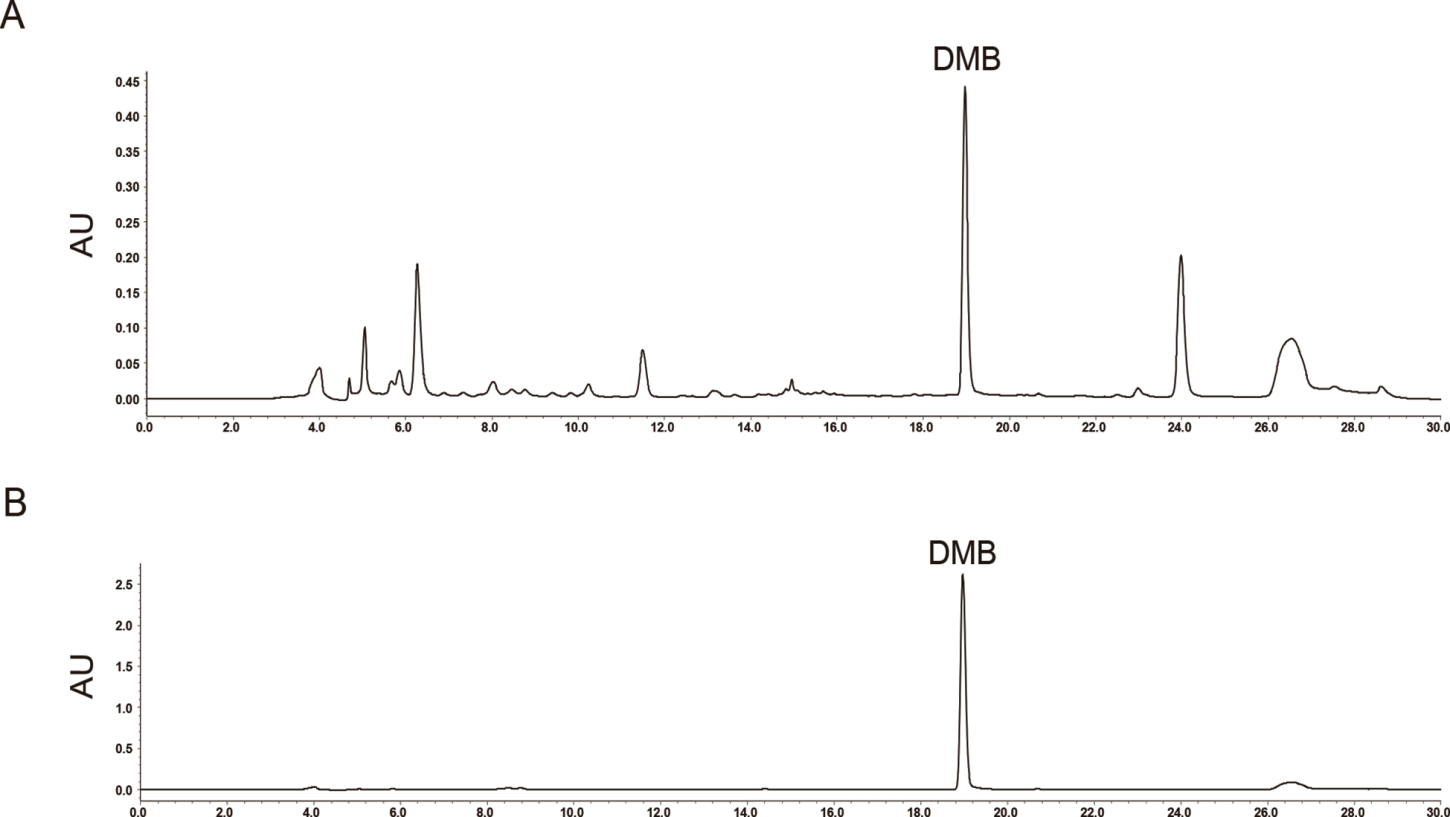
High-performance liquid chromatography (HPLC) of *Gentianella acuta* extract (GAE) and its standard marker substance. **(A)** HPLC of GAE recorded at 254 nm and peak of desmethylbellidifolin (DMB) is shown. **(B)** Standard peak of DMB is shown.

Content of DMB in GAE was quantified to be 4.93 mg/g by HPLC, with a correlation coefficient *r* > 0.9997, and an average recovery of 97.84% (RSD = 1.1%, *n* = 6) (**Figure 1**).

### GAE and DMB Alleviates TNBS-Induced UC

To investigate the physiological relevance of GAE and DMB-regulated suppression of colitis inflammatory process *in vivo*, a TNBS model of colitis was used. In the course of the experiment, rats were monitored daily for weight loss, food weight, water volume, as well as signs of rectal bleeding and diarrhea.

After 10 consecutive days of GAE (100, 200, and 400 mg/kg) oral administration, compared to control group, body weights were significantly increased in GAE-treated groups. Symptoms of UC, such as bloody stools and diarrhea, were ameliorated. Disease activity index (DAI), an index of UC physician’s rating of disease activity, was significantly improved by GAE treatment (**Supporting Figure S1**).

**Figure 2:**
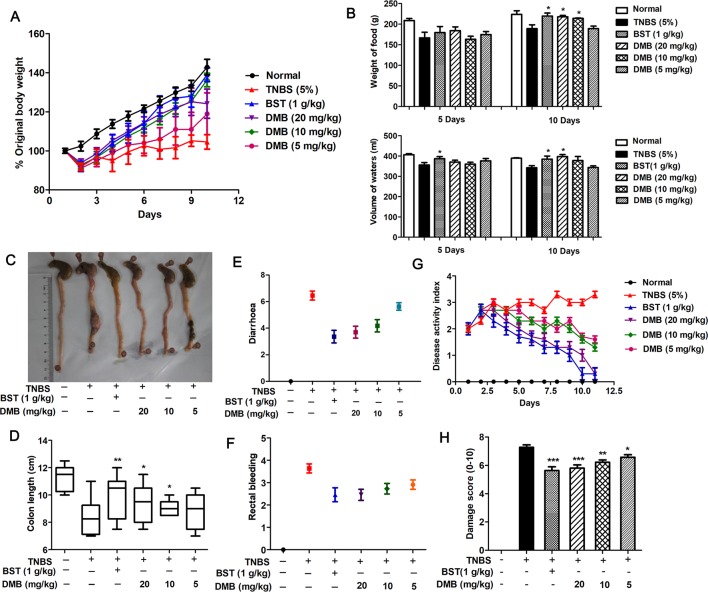
Desmethylbellidifolin (DMB) alleviates trinitrobenzenesulfonic acid (TNBS)-induced ulcerative colitis. Rat were injected with TNBS through the anus. After 24 h, DMB was administrated orally in 5% gum acacia at 5, 10, and 20 mg/kg BW, respectively, for the DMB group, while the Balsalazide sodium tablets (BST) group and model group received the same volume of 5% acacia water solution with or without 1 g/kg BST for consecutive 10 days. The indicators were measured body weight **(A)**, weight of food, and volume of waters **(B)**. Colons were isolated and photographed **(C)**. Colon lengths were measured **(D)**. Diarrhea score **(E)**, rectal bleeding **(F)**, disease activity index **(G)**, and damage score **(H)** were calculated. Data are presented as mean ± SD, and *, **, and *** indicate *P* < 0.05, *P* < 0.01, and *P* < 0.001, respectively.

To further confirm the role of GAE active constituent in UC treatment, DMB, was tested using the same model. The results showed that TNBS treatment caused markedly weight loss in the model group, whereas mice treated with DMB were dramatically protected from weight loss and colon shortening in a dose-dependent manner (**Figures 2A, C, D**). Consistently, a similar trend was found in food and water intake (**Figure 2B**). Moreover, as shown in **Figure 2G**, DMB treatment ameliorated TNBS-stimulated DAI. We found that DMB treatment significantly inhibited TNBS-induced rectal bleeding and diarrhea and reduced TNBS-induced production of bloody stools and damage score (**Figures 2E, F, H**).

**Figure 3:**
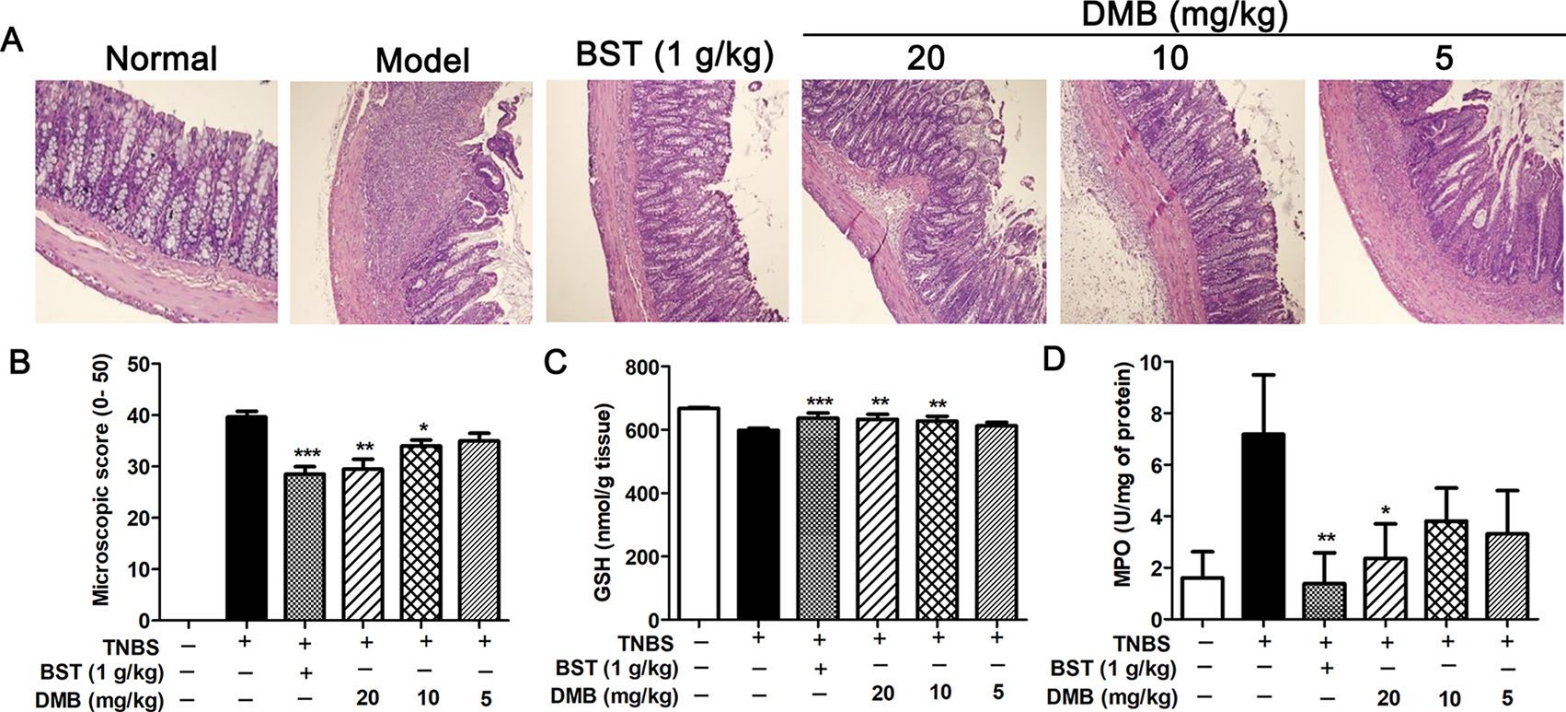
Desmethylbellidifolin (DMB) improve colorectal histology in trinitrobenzenesulfonic acid (TNBS)-induced acute colitis rat model. Representative HE staining image **(A)**. Microscopic score **(B)**. Colonic glutathione (GSH) and myeloperoxidase (MPO) levels **(C** and **D)**. Data are representative of four separate experiments with similar results. Data are presented as mean ± SD, and *, **, and *** indicate *P* < 0.05, *P* < 0.01, and *P* < 0.001, respectively.

Next, we measured the effects of DMB on colorectal histology in rat with TNBS-induced colitis. As shown in **Figure 3A**, DMB treatment showed the alleviated morphological alteration and lower inflammation level with scattered infiltration of monocytes in TNBS-treated rat. Compared to the Model group, DMB treatment could protect the intestinal integrity in all colonic layers (**Figure 3B**). In addition, the glutathione level was induced by DMB administration in the colon (**Figure 3C**). The MPO activity is a marker of neutrophil infiltration, which plays an extremely important role in polymorphonuclear infiltration. As shown in **Figure 3D**, DMB treatment significantly inhibited TNBS-induced MPO activity. Taken together, these results suggested that DMB reduced TNBS-induced acute colitis.

### GAE and DMB Inhibits Mice Intestine Smooth Muscle Contraction

**Figure 4:**
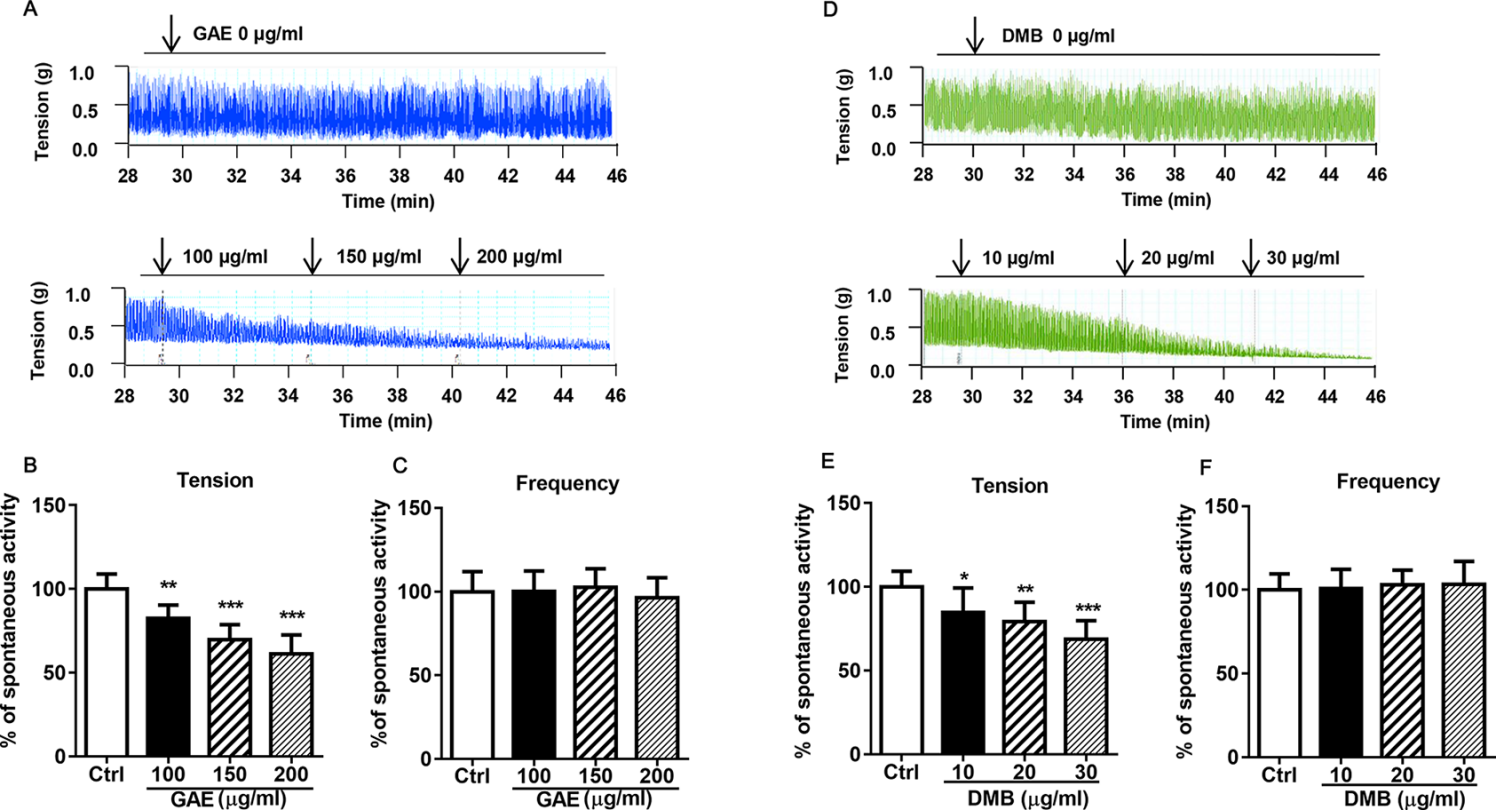
*Gentianella acuta* extract (GAE) and desmethylbellidifolin (DMB) inhibit mice intestine smooth muscle spontaneous contraction. The isolated distal colonic strips were longitudinally mounted in an organ bath, and the contractile response to GAE or DMB was recorded. Active tension on spontaneous contractions of intestine smooth muscle **(A**, **B** and **D**, **E)**. Active frequency on spontaneous contractions of intestine smooth muscle **(C** and **F)**. Data are expressed as means ± SD (*n* = 7); * and *, **, and *** indicate *P* < 0.05, *P* < 0.01, and *P* < 0.001, respectively.

We investigated the effects of GAE and DMB on intestinal smooth muscle spontaneous contraction. As shown in **Figure 4**, both GAE (100, 150, and 200 µg/ml) and DMB (10, 20, and 30 µg/ml) prominently inhibited the active tension on intestine smooth muscle contractions in a concentration-dependent manner. However, active frequency was not altered by the GAE or DMB treatments.

### DMB Promotes the Recovery of KCl-, Ach-, BaCl_2_-, or HA-Induced Intestine Smooth Muscle Contraction

**Figure 5:**
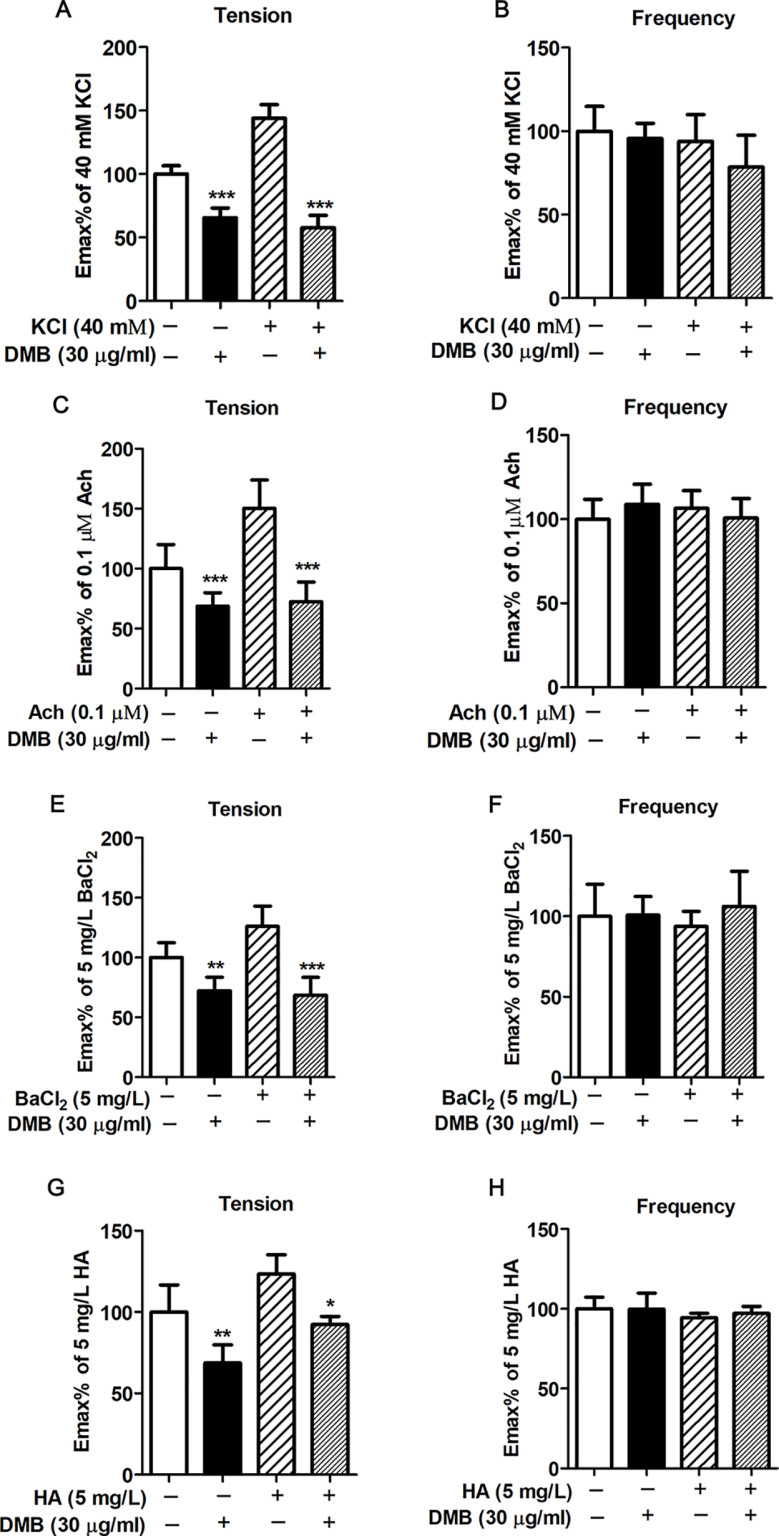
Desmethylbellidifolin (DMB) promotes the recovery of KCl-, ACh-, BaCl_2_-, or HA-induced intestine smooth muscle contraction. After whole distal colonic strips were longitudinally mounted in an organ bath, KCl, ACh, BaCl_2_, or HA was treated for 5 min, followed by treatment of DMB for 10 min, respectively. The contractility was measured by a tonotransducer. The percentage change of active tension **(A**, **C**, **E**, **G)** and active frequency **(B**, **D**, **F**, **H)** of contractile response were recorded. Data are expressed as means ± SD (*n* = 7); * and *, **, and *** indicate *P* < 0.05, *P* < 0.01, and *P* < 0.001, respectively.

To further determined the spasmolytic activity of DMB, spasmogens, such as KCl, ACh, BaCl_2_, or HA, induced intestinal smooth muscle contraction models were used. As shown in **Figure 5** and **S2**, the KCl-, ACh-, BaCl_2_-, or HA-induced intestine smooth muscle contraction was dramatically suppressed by DMB, while the active frequency was unaffected.

### DMB Inhibits TNBS-Induced Inflammation in Mice Colon

**Figure 6:**
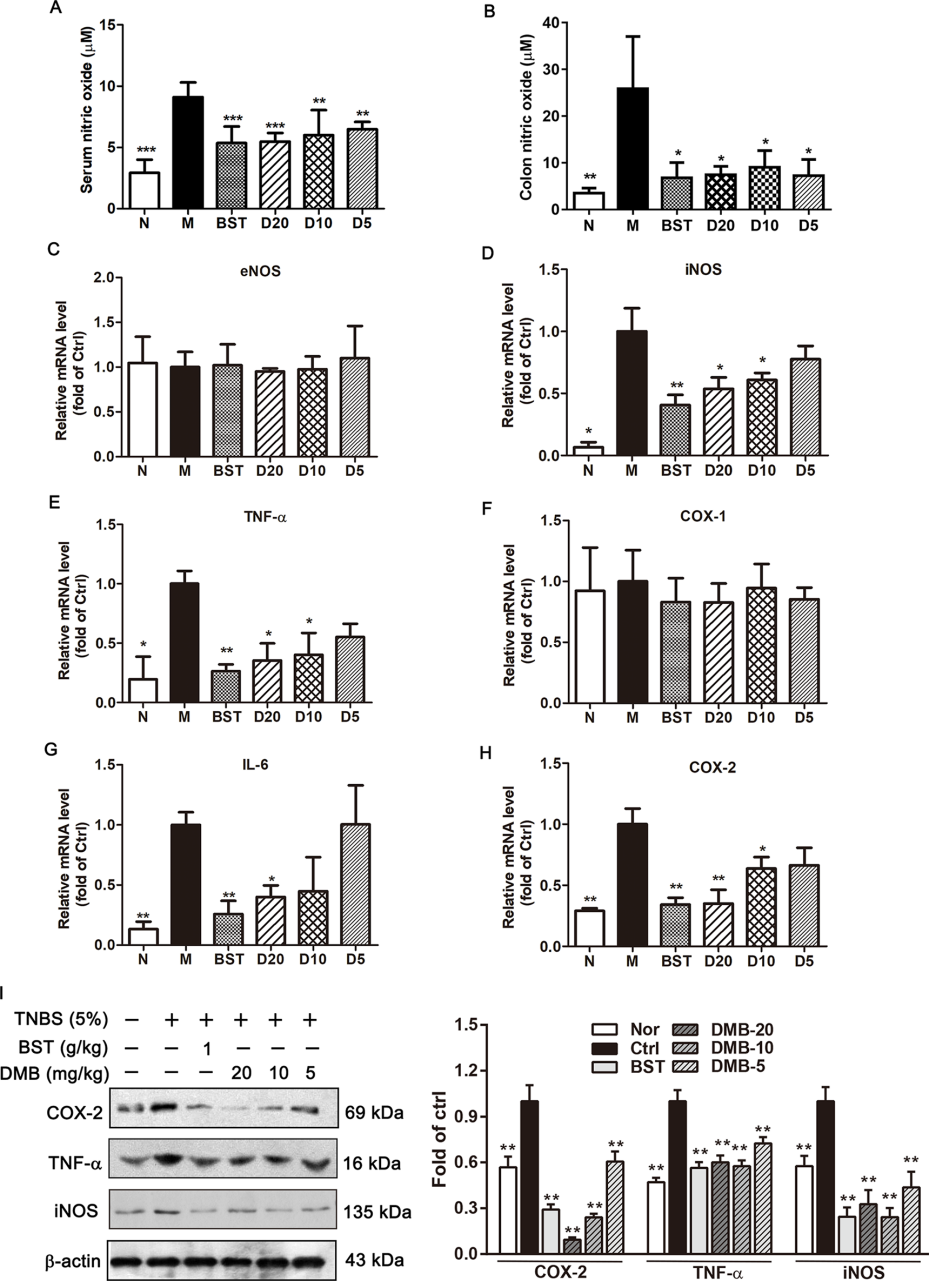
Desmethylbellidifolin (DMB) inhibits trinitrobenzenesulfonic acid (TNBS)-induced inflammation in rat colon. Rat were injected with TNBS through anus. After 24 h, DMB was administrated orally in 5% gum acacia at 5, 10, and 20 mg/kg BW, respectively (D5, D10, and D20) for the DMB group. while the BST group and model group (M) received the same volume of 5% acacia water solution with or without 1 g/kg BST for consecutive 10 days. NO level in TNBS-induced colitis rat blood serum and colon tissue **(A** and **B)**. Messenger RNA (mRNA) expression of eNOS, iNOS, IL-6, TNF-α, COX-1, and COX-2 were determined by real-time reverse transcription PCR (RT-PCR) **(C**–**H)**. Cyclooxygenase 2 (COX-2), tumor necrosis factor-α (TNF-α), and nitric oxide synthase (iNOS) protein expression in TNBS-treated rat colon **(I)**. Data are expressed as means ± SD (*n* = 8); * and *, **, and *** indicate *P* < 0.05, *P* < 0.01, and *P* < 0.001, respectively.

To investigate whether the DMB has potential anti-inflammatory effects in TNBS-induced acute colitis, NO concentration was measured by Griess reagent in the presence or absence of DMB. As shown in **Figures 6A, B**, NO production was strongly induced in TNBS-stimulated acute colitis, while pretreatment with DMB notably inhibit the TNBS-induced NO production. We also determined whether DMB could inhibit TNBS-induced proinflammatory cytokines, endothelial nitric oxide synthase (eNOS), iNOS, TNF-α, COX-1, interleukin-6 (IL-6), and COX-2 gene expression. As shown in **Figures 6D, E, G, H**, DMB significantly decreased the messenger RNA (mRNA) levels of iNOS, IL-6, TNF-α, and COX-2 in a concentration-dependent manner. However, there was no obvious difference in the mRNA levels of eNOS and COX-1 between the model and DMB-treated groups (**Figures 6C, F**).

### DMB Inhibits TNF-α, iNOS, and COX-2 Protein Expression *in Vitro* and *in Vivo*

**Figure 7:**
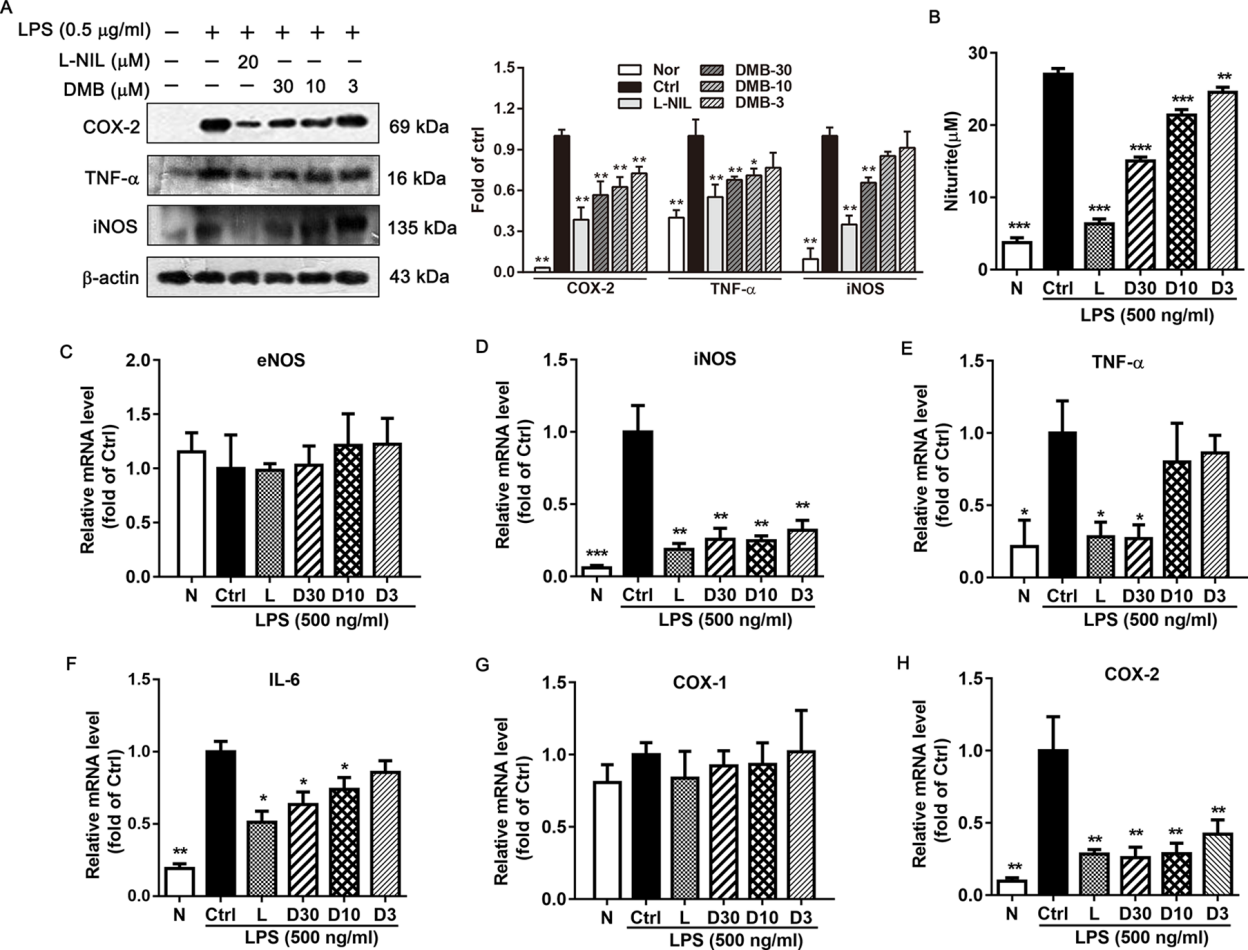
Desmethylbellidifolin (DMB) inhibits lipopolysaccharides (LPS)-induced inflammatory cytokines expression in RAW264.7 cells. Cells were divided as the following groups: N (normal), Ctrl (control), L (20 µM L-N6-1-iminoethyl-lysine), or D (30, 10, and 3 µM DMB). Cyclooxygenase 2 (COX-2), tumor necrosis factor-α (TNF-α), and nitric oxide synthase (iNOS) protein expression in LPS-induced RAW264.7 cells **(A)**. NO level in LPS-induced RAW264.7 cells **(B)**. Messenger RNA (mRNA) expression of endothelial nitric oxide synthase (eNOS), iNOS, interleukin-6 (IL-6), TNF-α, COX-1, and COX-2 were determined by real-time RT-PCR **(C**–**H)**. Data are expressed as means ± SD (*n* = 3); * and *, **, and *** indicate *P* < 0.05, *P* < 0.01, and *P* < 0.001, respectively.

The iNOS and COX-2 are often involved in the induction of inflammatory and malignant conditions. Thus, iNOS and COX-2 protein expression levels were determined. As shown in **Figures 6I** and **7A**, the expression level of iNOS and COX-2 were significantly elevated by TNBS/LPS treatment *in vitro* and *in vivo*. Consistent with mRNA results, the expression of iNOS and COX-2 were dramatically suppressed by the DMB treatment in a dose-dependent manner. Moreover, TNF-α protein level was also decreased by the DMB.

### DMB Inhibits LPS-Induced Inflammatory Cytokine Expression in RAW264.7 Cells

To further confirm the anti-inflammatory effects of DMB, we determined inflammatory cytokine expression in LPS-stimulated RAW264.7 cells. As shown in **Figure 7B**, DMB treatment could inhibit LPS-stimulated NO production in Raw264.7 cells. in addition, mRNA expression levels of iNOS, TNF-α, IL-6, and COX-2 were significantly decreased by DMB (**Figures 7D–F, H**), while DMB had little effects on eNOS and COX-1 mRNA expression level (**Figures 7C, G**).

## Discussion

In this study, we demonstrated the anti-UC role of GAE and its xanthone compound DMB and investigated the potential mechanisms both *in vivo* and *in vitro*. Our novel findings include the following: 1) We identified DMB, a natural xanthone from GAE, as an active substance on UC treatment; 2) we found that GAE and DMB not only suppressed the symptom and histopathological changes of UC but also exert an antispasmodic effect; and 3) mechanically, we showed that the protective effect of GAE and DMB on UC may partially through its inhibitory effect on proinflammation cytokine expression and NO production.

UC is a chronic and relapsing inflammatory disorder of the gastrointestinal tract disease. The common clinical symptom of UC is abdominal cramping, bloody diarrhea, and bloating with a change in stool frequency and form. These symptoms can be embarrassing, uncomfortable, and affect the quality of life. Therefore, it is important to develop therapeutic approaches to relieve the symptoms of UC (Abraham, 2015). Here, we showed that GAE and DMB treatment could ameliorate TNBS-induced rat model weight loose and food/water intake decrease, increase colon length, and attenuate DAI and histopathological score. Furthermore, histological studies revealed that DMB reduced TNBS-induced pathological lesions, suggesting protective role of DMB in colon inflammation pathophysiology. These results indicated that DMB has symptomatic relief effect for UC-caused abdominal discomfort.

Inflammation is the key factor in UC occurrence and development. Chronic inflammation increases gut epithelial permeability, leading to the system exposure to intestine bacterial endotoxin products, such as LPS, and aggravate inflammation condition (Kawashima et al., 2011; Funderburg et al., 2013). In UC patient, serum levels of the proinflammatory cytokines significantly increased, including TNF-α, IL-1β, IL-6, etc. (Rogler, 2017). TNF-α can activate nuclear factor kappa B (NF-κB), and its expression is induced in response to NF-κB activation, thus forming an amplifying feed forward loop. NF-κB activation could also induce the expression of iNOS and COX-2. The disequilibrium between anti- and proinflammatory cytokines resulting in hyperresponsive state of the UC (Kim and Cheon, 2017). Recently, various anticytokines and adhesion drugs have been developed for the treatment of UC patients. Some anti-TNF-α medicines were used to block part of the process that causes inflammation in the body, such as Natalizumab, a new antibody for treatment of UC (Baumgart and Sandborn, 2007). However, the side effect and intravenous administration approach limited its use in patients. It has been reported that GAE could significantly inhibit NF-κB activation (Li et al., 2019). Indeed, our results showed that administration of DMB reduced NO level in serum and colon tissue in TNBS-induced UC model and suppressed the TNF-α, iNOS, and COX-2 expression both *in vivo* and *in vitro*. These findings suggested that DMB may act through inhibiting NF-κB signaling pathway to inhibit the inflammation in intestine and alleviate UC development. Abnormal intestinal contraction is one of the causes of UC-induced abdominal pain or discomfort. In UC patients with abdominal pain and diarrhea, colony motility index and high-amplitude-propagating contractions were significantly greater than in healthy volunteer. It has been reported that the levels of 5-hydroxytryptamine (5-HT) and M and H receptors and their corresponding ligands are significantly higher in UC patients than those in the healthy controls (Huang et al., 2005; Ohama et al., 2007). These receptors play functional roles in intestinal smooth muscle contraction through regulating intracellular Ca^2+^ concentration. Medications targeting these receptors and their signaling pathways have been developed for UC symptom management. Alosetron (5-HT3 antagonist), Darifenacin (a selective muscarinic M3 receptor inhibitor), and ebastine (a selective H1 receptor inhibitor) have been used to reduce the colonic contractile response and abdominal pain in UC (Bharucha et al., 2010; Abraham, 2015). However, combination of medications causes inconvenience and increases the possibility of noncompliance. KCl, ACh, histamine, and BaCl2 can either activate these receptors or promote intracellular Ca^2+^ concentration accumulation, thereby inducing intestinal contraction. In this study, we investigated the inhibitory effect of DMB on KCl-, ACh-, histamine-, and BaCl_2_-induced contractile response in isolated intestinal model. The results showed that DMB caused a significant decrease in abnormal intestinal contraction in the four experimental conditions. The results indicated that DMB has an antispasmodic effect by targeting major calcium-dependent receptors to alleviate the UC-caused abnormal intestinal contraction.

UC is characterized by a gastrointestinal functional disorder with multiple pathogenic mechanisms and is not likely to result from one single-target defect. Although some single-target medications, such as aminosalicylates, corticosteroids, infliximab, and azathioprine, were used for UC that were focused on relieving symptoms and preventing or managing chronic inflammatory conditions, single use of anti-inflammatory drug does not meet clinic requirement in all people, and some can cause serious side effects. In addition, it is difficult to organize a perfect dosage ratio for different kinds of medicines to obtain an optimal therapeutic effect.

It has become more and more evident that a multiple-target integrative medicine is superior to the single-target drugs for chronic complex disease treatment. Considering the chronic nature of UC and side effects of conventional therapies, integrative multitarget drug intervention is considered more efficient in modulating networks than targeting a single macromolecule with a high-affinity ligand (Koeberle and Werz, 2014; Misra, 2014). As a natural xanthone, DMB has nonselective multitarget property for UC intervention. Here, we showed that it has more active anti-inflammatory and antispasmodic effects than positive control agents, which suggest the potential use of DMB for the UC treatment in clinic.

In summary, our results showed that DMB could reduce inflammatory response and alleviate colon muscle spasm. DMB may be a good candidate for subsequent development as a multitargeting drug for treatment of UC.

## Data Availability

The raw data supporting the conclusions of this manuscript will be made available by the authors, without undue reservation, to any qualified researcher.

## Ethics Statement

All animal experiments were approved by Tianjin University of Traditional Chinese Medicine Committee on Use and Care of Animals (TCM-LAEC20170037).

## Author Contributions

ML, YZ, and TW contributed to experimental design. YN, ML, HY, YC, YL, and JR contributed to the acquisition and analysis of data. HY, SC, AD, and TW reviewed the manuscript. TW acquired the funding. ML and TW wrote the manuscript.

## Funding

This work was supported by National Natural Science Foundation of China (81673688, 81673703), Important Drug Development Fund, Ministry of Science and Technology of China (2018ZX09735002), and the Research Program of Tianjin Municipal Education Commission (2017KJ129).

## Conflict of Interest Statement

The authors declare that the research was conducted in the absence of any commercial or financial relationships that could be construed as a potential conflict of interest.

## References

[B1] Abraham B. P. (2015). Symptom management in inflammatory bowel disease. Expert Rev. Gastroenterol. Hepatol. 9 (7), 953–967. 10.1586/17474124.2015.1038241 25905569

[B2] Alex P. Zachos N. C. Nguyen T. Gonzales L. Chen T. E. Conklin L. S. (2009). Distinct cytokine patterns identified from multiplex profiles of murine DSS and TNBS-induced colitis. Inflamm. Bowel Dis. 15 (3), 341–352. 10.1002/ibd.20753 18942757 PMC2643312

[B3] Baumgart D. C. Sandborn W. J. (2007). Gastroenterology 2—Inflammatory bowel disease: clinical aspects and established and evolving therapies. Lancet 369 (9573), 1641–1657. 10.1016/S0140-6736(07)60751-X 17499606

[B4] Bell C. J. Gall D. G. Wallace J. L. (1995). Disruption of colonic electrolyte transport in experimental colitis. Am. J. Physiol. 268 (4 Pt 1), G622–G630. 10.1152/ajpgi.1995.268.4.G622 7733288

[B5] Bharucha A. E. Ravi K. Zinsmeister A. R. (2010). Comparison of selective M3 and nonselective muscarinic receptor antagonists on gastrointestinal transit and bowel habits in humans. Am. J. Physiol. Gastrointest. Liver Physiol. 299 (1), G215–G219. 10.1152/ajpgi.00072.2010 20395537 PMC2904119

[B6] Blumberg R. Cho J. Lewis J. Wu G. (2011). Inflammatory bowel disease: an update on the fundamental biology and clinical management. Introduction. Gastroenterology 140 (6), 1701–1703. 10.1053/j.gastro.2011.03.013 21530735

[B7] Fiocchi C. (1998). Inflammatory bowel disease: etiology and pathogenesis. Gastroenterology 115 (1), 182–205. 10.1016/S0016-5085(98)70381-6 9649475

[B8] Funderburg N. T. Park S. R. S. Sung H. C. Hardy G. Clagett B. Ignatz-Hoover J. (2013). Circulating CD4(+) and CD8(+) T cells are activated in inflammatory bowel disease and are associated with plasma markers of inflammation. Immunology 140 (1), 87–97. 10.1111/imm.12114 23600521 PMC3809709

[B9] Herfarth H. H. Martin C. F. Sandler R. S. Kappelman M. D. Long M. D. (2014). Prevalence of a gluten-free diet and improvement of clinical symptoms in patients with inflammatory bowel diseases. Inflamm. Bowel Dis. 20 (7), 1194–1197. 10.1097/MIB.0000000000000077 24865778 PMC4331053

[B10] Huang J. Zhou H. Mahavadi S. Sriwai W. Lyall V. Murthy K. S. (2005). Signaling pathways mediating gastrointestinal smooth muscle contraction and MLC20 phosphorylation by motilin receptors. Am. J. Physiol. Gastrointest. Liver Physiol. 288 (1), G23–G31. 10.1152/ajpgi.00305.2004 15591586

[B11] Kaplan G. G. Ng S. C. (2017). Understanding and preventing the global increase of inflammatory bowel disease. Gastroenterology 152 (2), 313–321 e312. 10.1053/j.gastro.2016.10.020 27793607

[B12] Katz S. Pardi D. S. (2011). Inflammatory bowel disease of the elderly: frequently asked questions (FAQs). Am. J. Gastroenterol. 106 (11), 1889–1897. 10.1038/ajg.2011.271 21862997

[B13] Kawashima R. Kawamura Y. I. Oshio T. Son A. Yamazaki M. Hagiwara T. (2011). Interleukin-13 damages intestinal mucosa *via* TWEAK and Fn14 in mice—a pathway associated with ulcerative colitis. Gastroenterology 141 (6), 2119–2129 e2118. 10.1053/j.gastro.2011.08.040 21893119

[B14] Kim D. H. Cheon J. H. (2017). Pathogenesis of inflammatory bowel disease and recent advances in biologic therapies. Immune Netw. 17 (1), 25–40. 10.4110/in.2017.17.1.25 28261018 PMC5334120

[B15] Koeberle A. Werz O. (2014). Multi-target approach for natural products in inflammation. Drug Discov. Today 19 (12), 1871–1882. 10.1016/j.drudis.2014.08.006 25172801

[B16] Li A. Y. Wang J. J. Yang S. C. Zhao Y. S. Li J. R. Liu Y. (2019). Protective role of *Gentianella acuta* on isoprenaline induced myocardial fibrosis in rats *via* inhibition of NF-kappaB pathway. Biomed. Pharmacother. 110, 733–741. 10.1016/j.biopha.2018.12.029 30554111

[B17] Liu M. Zhang W. Li X. Han J. Chen Y. Duan Y. (2016a). Impact of age and sex on the development of atherosclerosis and expression of the related genes in apoE deficient mice. Biochem. Biophy. Res. Commun. 469 (3), 456–462. 10.1016/j.bbrc.2015.11.064 26592663

[B18] Liu Y. Ni Y. Ruan J. Qu L. Yu H. Han L. (2016b). Bioactive gentixanthone and gentichromone from the whole plants of *Gentianella acuta* (Michx.) Hulten. Fitoterapia 113, 164–169. 10.1016/j.fitote.2016.08.001 27514655

[B19] Liu Y. Li C. Shi X. Li P. Ying W. Li B. (2015). Determination of bellidifolin and norswertianolin from *Gentianella acuta* by HPLC. Chin. Arch. Tradit. Chin. Med. 33 (9), 2246–2248. 10.13193/j.issn.1673-7717.2015.09.055

[B20] Loening-Baucke V. Metcalf A. M. Shirazi S. (1989). Anorectal manometry in active and quiescent ulcerative colitis. Am. J. Gastroenterol. 84 (8), 892–897. 2756980

[B21] Misra S. M. (2014). Integrative therapies and pediatric inflammatory bowel disease: the current evidence. Children (Basel, Switzerland) 1 (2), 149–165. 10.3390/children1020149 PMC4928727 27417473

[B22] Morris G. P. Beck P. L. Herridge M. S. Depew W. T. Szewczuk M. R. Wallace J. L. (1989). Hapten-induced model of chronic inflammation and ulceration in the rat colon. Gastroenterology 96 (3), 795–803. 10.1016/0016-5085(89)90904-9 2914642

[B23] Ohama T. Hori M. Ozaki H. (2007). Mechanism of abnormal intestinal motility in inflammatory bowel disease: how smooth muscle contraction is reduced? J. Smooth Muscle Res. 43 (2), 43–54. 10.1540/jsmr.43.43 17598957

[B24] Rogler G. (2017). Resolution of inflammation in inflammatory bowel disease. Lancet Gastroenterol. Hepatol. 2 (7), 521–530. 10.1016/S2468-1253(17)30031-6 28606878

[B25] Schirbel A. Fiocchi C. (2010). Inflammatory bowel disease: established and evolving considerations on its etiopathogenesis and therapy. J. Dig. Dis. 11 (5), 266–276. 10.1111/j.1751-2980.2010.00449.x 20883422

[B26] Singh S. Feuerstein J. D. Binion D. G. Tremaine W. J. (2018). American Gastroenterological Association technical review on the management of mild-to-moderate ulcerative colitis. Gastroenterology 156 (3), 769–808. 10.1053/j.gastro.2018.12.008 30576642 PMC6858923

[B27] Swidsinski A. Ladhoff A. Pernthaler A. Swidsinski S. Loening-Baucke V. Ortner M. (2002). Mucosal flora in inflammatory bowel disease. Gastroenterology 122 (1), 44–54. 10.1053/gast.2002.30294 11781279

[B28] Verma S. Brown S. Kirkwood B. Giaffer M. H. (2000). Polymeric versus elemental diet as primary treatment in active Crohn’s disease: a randomized, double-blind trial. Am. J. Gastroenterol. 95 (3), 735–739. 10.1016/S0002-9270(99)00586-9 10710067

[B29] Yang Y. Yan H. Jing M. Zhang Z. Zhang G. Sun Y. (2016). Andrographolide derivative AL-1 ameliorates TNBS-induced colitis in mice: involvement of NF-small ka, CyrillicB and PPAR-gamma signaling pathways. Sci. Rep. 6, 29716. 10.1038/srep29716 27435110 PMC4951727

[B30] Zhang Y. Liu X. Han L. Gao X. Liu E. Wang T. (2013). Regulation of lipid and glucose homeostasis by mango tree leaf extract is mediated by AMPK and PI3K/AKT signaling pathways. Food Chem. 141 (3), 2896–2905. 10.1016/j.foodchem.2013.05.121 23871039

