# Supplementary material for: Desmethylbellidifolin From Gentianella acuta Ameliorate TNBS-Induced Ulcerative Colitis Through Antispasmodic Effect and Anti-Inflammation
Source: Front Pharmacol. 2019 Sep 20;10:1104. doi: 10.3389/fphar.2019.01104 (PMC6764246; doi:10.3389/fphar.2019.01104)
Supplement: Supplementary file 1 [file DataSheet_1.docx]

**
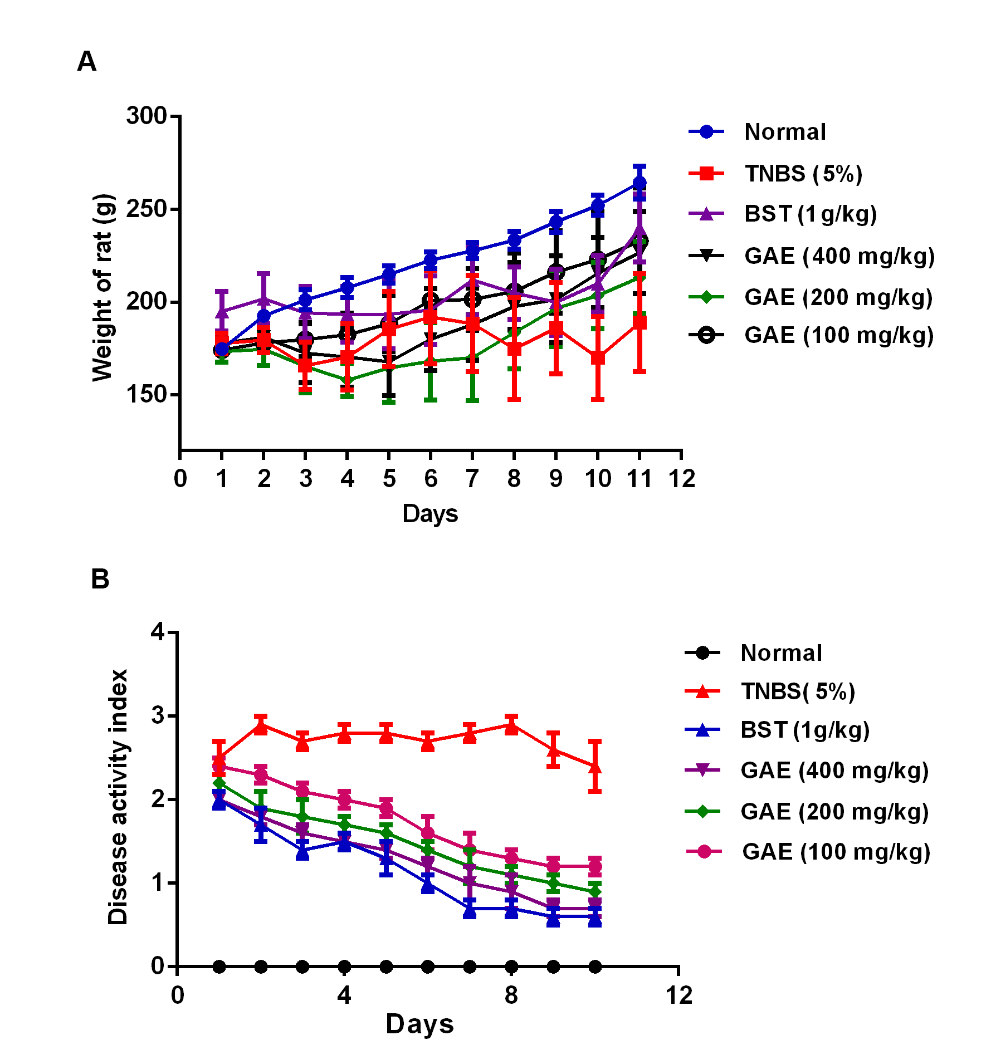
Supporting Figures**

**Figure S1.** GAE alleviates TNBS-induced ulcerative colitis. Rat were injected with TNBS through anus. After 24 h, GAE was administrated orally in 5% gum acacia for DMB group. while the BST group and control group received same volume of 5% acacia water solution with or without 1 g/kg BST for consecutive 10 days. The body weight (A) and disease activity index (B) were determined.


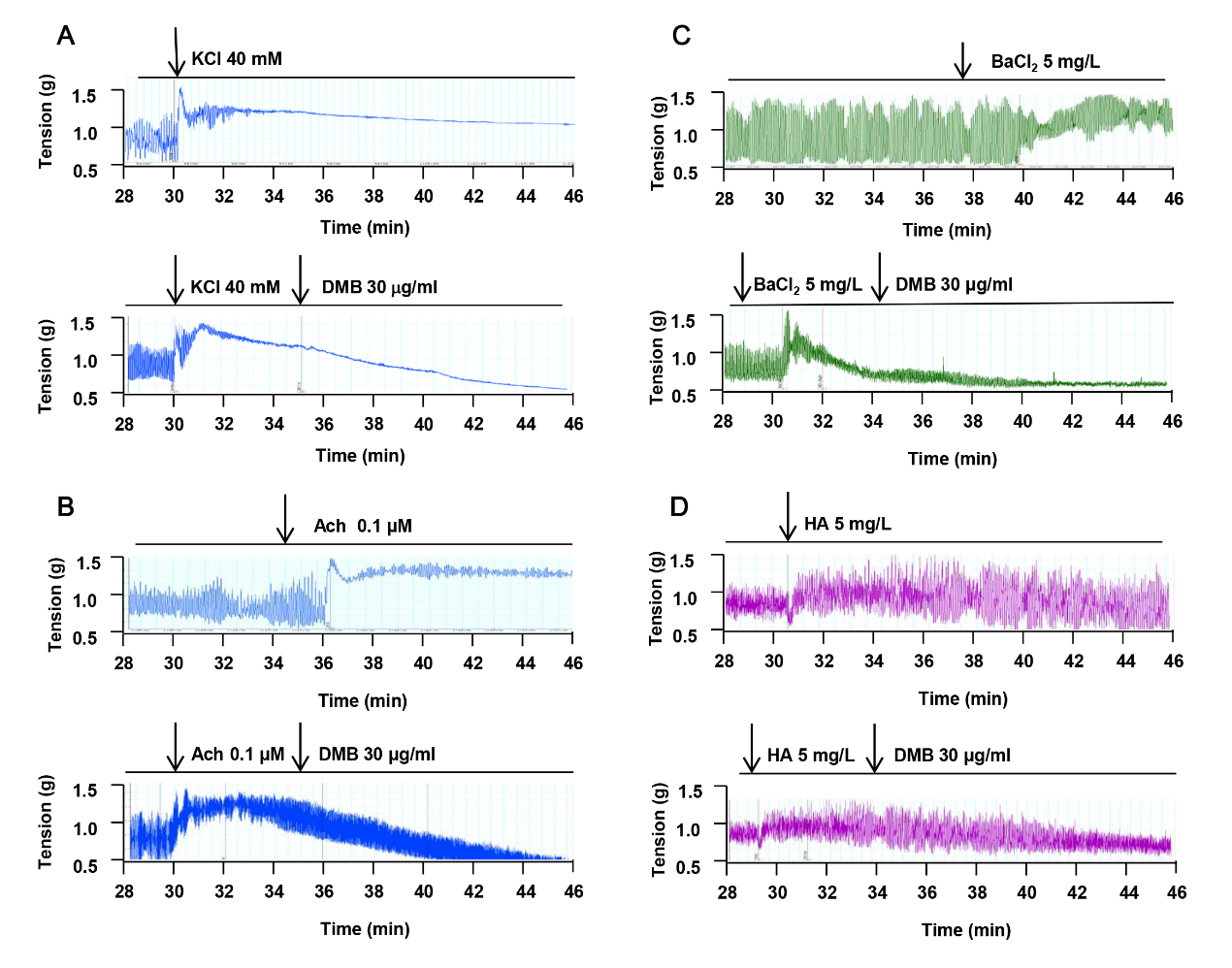
**Figure S2.** Contractile patterns produced by DMB treatment in KCl, ACh, BaC_l2_ or HA pre-treated longitudinal muscle of distal colon. After whole distal colonic strips were longitudinally mounted in an organ bath, KCl (A), ACh (B), BaCl_2_ (C) or HA (D) was treated for 5 min, followed by treatment of DMB for 10 min respectively. The contractility was measured by tonotransducer and representative tracings were showed.
